# Optimization of deficit irrigation and nitrogen fertilizer management for peanut production in an arid region

**DOI:** 10.1038/s41598-021-82968-w

**Published:** 2021-03-09

**Authors:** Vijay Singh Rathore, Narayan Singh Nathawat, Seema Bhardwaj, Bhagirath Mal Yadav, Mahesh Kumar, Priyabrata Santra, Praveen Kumar, Madan Lal Reager, Narendra Dev Yadava, Om Parkash Yadav

**Affiliations:** 1 ICAR- Central Arid Zone Research Institute, Regional Research Station, Bikaner, 334004 India; 2 ICAR- Central Arid Zone Research Institute, Jodhpur, 342003 India; 3 KVK, Swami Keshwanand Rajasthan Agricultural University, Bikaner, 334006 India

**Keywords:** Plant sciences, Plant physiology

## Abstract

Deficit irrigation (DI) has been emerging as an important technique for enhancing crop water productivity (WP). However, advantage of DI under varying nitrogen (N) application rates remains unclear. Field experiments were conducted during 2012–2014 to investigate the impacts of six irrigation levels[FI (full irrigation), DI_10_, DI_20_, DI_30_, DI_40_ and DI_50_, with irrigation amount of 100, 90, 80, 70, 60 and 50% of ETc, respectively) and four N application rates (N_0_, N_10_, N_20_ and N_30_, with 0, 10, 20 and 30 kg N ha^−1^, respectively) on WP, yield, quality, and net economic return of peanut in hot arid region of India. We used Technique for Order Preference by Similarity to an Ideal Solution (TOPSIS) method to obtain the optimal combination of irrigation and N rates. Both irrigation level and nitrogen dose had significant effects on yield and quality parameters examined in the study. Relative to FI, DI_40_ and DI_50_ significantly reduced yield (40.2–62.1%), economic benefit (70.8–118.5%), WP (8.2–33.0%), and kernel oil content (7.5–11.9%), but DI_20_ increased WP by 17.1% with only marginal reduction in economic benefit (2.6%), and yield (3.2%). Compared to N_0_, the N_30_ had 1.7, 1.1, and 1.6-folds increased yield, oil content in the kernel, and WP, respectively. Among all treatments, DI_0_N_30_ had the greatest yield and net return; DI_20_N_30_ had greatest WP and oil content in the kernel. TOPSIS analysis showed that DI_20_N_30_ was optimal in balancing of WP, yield, net return, and quality of peanut in northwestern arid India. The results have direct implications for improving irrigation water and N management for irrigated crops in arid regions.

Demand for agricultural products will further increase^1^ to satisfy the needs of an increasing population. However, the availability of water for agriculture has been declining due to an increasing demand of water for non-agricultural sectors^1,2^. Shortage of irrigation water is seriously affecting agricultural production particularly in arid and semi-arid regions because irrigated agriculture is required for agricultural production in these regions. In the light of diminishing water resources for agriculture and increasing demand for crop production, irrigation strategies need to be devised to maximize crop water productivity (WP)^3^. Deficit irrigation (DI) which involves an application of the amount of irrigation water lesser than the full crop evapotranspiration (ET) is emerging as an important technique to enhance WP^4,5^. It had been reported that DI increased WP with considerable saving of irrigation water in many crops particularly in arid and semi-arid regions^6–9^*.* Adu et al*.*^10^ reported huge variations in yield response of different crops while analysing relative yield performances of 43 crops grown in 14 countries under DI and full irrigation (FI) suggesting that DI require crop and region specific information on suitable magnitude of irrigation deficit.Thus, implementation of DI requires crop specific information related to identification of suitable magnitude of irrigation deficit, and agronomic management for its effective use^11^.

The crop yield and WP are affected by climate, crop species, soil, crop management practices, and choice of cultivar^12–14^. Li et al*.*^14^ reported that agronomic practices influenced WP more than climatic factors; and from among various agronomic management, fertilizer rate and irrigation contributed 42.3% and 32.8%, respectively to the increase of WP. Soil nutrients directly influence photosynthesis and improve utilization of water by crops^13,15^. Adequate nutrient management is an important determinant of WP. Nitrogen (N) is one of the most important limiting factors for crop production in many regions of world^16^, and efficiency of N-fertilizer is low (30–50%)^17^ which increases the cost of production and causes environmental problems including soil quality deterioration and water contamination. It has been reported that if the water and N management are properly used, a synergistic interaction between water and N on growth and yield may occur and may also increase in WP and NUE^18,19^.

Peanut (*Arachis hypogaea* L.) is a major legume crop in arid and semi-arid regions of the world^20^. Its seed is rich in oil (48–50%), protein (25–28%), carbohydrates (20–26%), and energy (546 kcal 100 g^−1^), and contains several minerals, vitamins, dietary fibers, phytosterols, flavonoids, and phenolic acids^21^. India produces 7.4 × 10^9^ kg of peanut from an area of 4.8 × 10^6^ ha with northwestern arid region being the major production area^22,23^. Large gap between precipitation (350 mm) and potential evapotranspiration (approximately 2100 mm) implies that irrigation is essential for peanut production in this region. Peanut growers apply excessive irrigation (600–700 mm) and N-fertilizer (30–40 kg N ha^−1^) to maximize yield. The average pod yield is about 3000 kg^−1^ in this region. The excessive use of irrigation and N fertilizer leads to reduction in WP and NUE, and economic benefits along with many environmental problems. Therefore, suitable irrigation and N-fertilizer regimes are needed for augmenting water and N-fertilizer use efficiencies for sustainable peanut production in northwestern arid India.

Previous research conducted on peanut mostly dealt with individual component of irrigation levels^20,24–27^ and N-fertilizer rate^28–30^ and focused on narrow range of criteria, e.g. yields, WP, and quality. However, effects of different DI levels with varying N rates on WP, yield, quality, and economic benefit remained unclear. The information on integrated use of DI and N rates relating to suitable irrigation and N fertilization rates to achieve high yield, economic benefit, and WP with better quality and saving of irrigation water is very limited. Therefore, objectives of this study were to investigate effects of DI on growth, yield components, yield, quality, economic benefit, WP and NUE of peanut under varying N application rates; and to identify optimal combination of DI and N rates that can simultaneously improve yield, quality, economic benefit and WP using Technique for Order Preference by Similarity to an Ideal Solution (TOPSIS). This paper aims to provide a scientific basis for efficient management of irrigation and N-fertilizer in the hot arid region of northwest India using 3-year field experiments and data analysis using multi-criteria optimization procedure. TOPSIS is an important technique of multiple attribute decision making^31^, which identifies positive and negative ideal solutions and ranking of alternatives based on the relative closeness to positive ideal solution^32^. It has been used to identify suitable irrigation and/ or nutrient application scheduling in different crop^31–33^.

## Results

### Amount of irrigation, AET and yield–water relationship

Amount of irrigation application varied from 330.6 mm to 591.2 mm during three seasons (Table 1). The DI_10_, DI_20_, DI_30_, DI_40_ and DI_50_ had 8.8, 17.6, 26.5, 35.3 and 44.1% reduction in amount of irrigation application compared to FI. The irrigation levels (I), N application rates (N) and their interaction (I × N) had significant (*P* < 0.05) effects on crop water use measured in terms of actual evapotranspiration (AET) (Table 1). The AET varied from 515.6 mm to 763.2 mm. The FIN_30_ had significantly higher AET than all other treatments. The AET declined with a decrease in irrigation; and averaged across years and N rates, the AET under DI_10_, DI_20_, DI_30_, DI_40_ and DI_50_ was decreased by 6.1, 15.9, 20.6, 24.1 and 31.0%, respectively than under FI. The N application enhanced AET; and effects of N application on AET varied with irrigation levels. The N_30_ under FI and DI_10_ had significantly (*P* < 0.05) greater AET than in other N rates. Under DI_40_ and DI_50_, the difference in AET among N_10_, N_20_ and N_30_ was not significant (*P* > 0.05), but these treatments had significantly greater AET than under N_0_.

**Table 1:** Amount of irrigation applied, actual evapotranspiration (AET), yield components and yields of peanut under different irrigation and nitrogen application rate treatments [Values are mean ± standard error, *n* = 9 (3 × 3; 3 growing seasons and 3 replications per treatment)].

Treatments	Irrigation amount (mm)	AET (mm)	Pod number (*n*. m^−2^)	Kernel number (*n*. m^−2^)	100-Kernel weight (g)	Biomass yield (kg ha^−1^)	Pod yield (kg ha^−1^)	Kernel yield (kg ha^−1^)	Pod harvest index	Shelling fraction
FIN_0_	591.2	748.9 ± 6.1c	247 ± 10ghi	360 ± 15hi	35.0 ± 0.7hi	6185 ± 313g	2172 ± 71f	1189 ± 59gh	0.354 ± 0.012i	0.546 ± 0.008kl
FIN_10_	591.2	751.3 ± 7.6c	314 ± 8d	509 ± 16 cd	38.5 ± 0.6e	7636 ± 288d	2731 ± 79d	1669 ± 63e	0.359 ± 0.008hi	0.611 ± 0.007fgh
FIN_20_	591.2	756.4 ± 7.1b	414 ± 12c	708 ± 22b	42.4 ± 0.6b	9791 ± 211b	3658 ± 62c	2378 ± 75c	0.374 ± 0.008efg	0.649 ± 0.009c
FIN_30_	591.2	763.2 ± 7.7a	482 ± 6a	846 ± 18a	45.2 ± 0.9a	10,475 ± 335a	4055 ± 75a	2676 ± 43a	0.389 ± 0.009 cd	0.661 ± 0.001b
DI_10_N_0_	539.1	701.0 ± 6.1g	251 ± 12ghi	373 ± 14gh	34.6 ± 0.6hi	6061 ± 286gh	2173 ± 61f	1209 ± 48gh	0.362 ± 0.012ghi	0.556 ± 0.004k
DI_10_N_10_	539.1	705.8 ± 7.3f	328 ± 15d	545 ± 26c	38.8 ± 0.7d	7669 ± 321d	2817 ± 71d	1747 ± 57de	0.369 ± 0.008fgh	0.620 ± 0.008f
DI_10_N_20_	539.1	710.6 ± 7.9e	419 ± 17bc	702 ± 29b	41.8 ± 1.1bc	9434 ± 289b	3619 ± 74c	2369 ± 63c	0.385 ± 0.008cde	0.655 ± 0.008bc
DI_10_N_30_	539.1	719.2 ± 7.9d	483 ± 13a	856 ± 24a	45.1 ± 0.5a	9657 ± 331b	3825 ± 78b	2553 ± 83ab	0.397 ± 0.007bc	0.670 ± 0.025a
DI_20_N_0_	487.0	627.9 ± 8.7i	227 ± 10ij	313 ± 12ij	34.3 ± 0.5i	5597 ± 288hi	2101 ± 64f	1149 ± 42h	0.379 ± 0.011def	0.547 ± 0.007kl
DI_20_N_10_	487.0	631.4 ± 7.7i	297 ± 12ef	446 ± 16ef	38.4 ± 0.4e	7129 ± 333ef	2749 ± 88d	1716 ± 77de	0.387 ± 0.008cde	0.623 ± 0.01e
DI_20_N_20_	487.0	639.7 ± 5.9h	399 ± 14c	653 ± 21b	40.7 ± 0.6c	8869 ± 330c	3550 ± 93c	2293 ± 74c	0.410 ± 0.009ab	0.647 ± 0.009c
DI_20_N_30_	487.0	640.5 ± 7.1h	447 ± 14b	798 ± 28a	44.2 ± 0.9a	8822 ± 315c	3810 ± 85b	2546 ± 63b	0.433 ± 0.009a	0.671 ± 0.017a
DI_30_N_0_	434.8	594.5 ± 7.5k	200 ± 9jk	262 ± 12jk	32.7 ± 0.5j	5345 ± 370i	1872 ± 45g	1016 ± 35i	0.358 ± 0.015hi	0.542 ± 0.005l
DI_30_N_10_	434.8	598.0 ± 7.1k	267 ± 17fgh	396 ± 24f-h	37.3 ± 0.6ef	6682 ± 403f	2484 ± 66e	1474 ± 59f	0.377 ± 0.012def	0.593 ± 0.008i
DI_30_N_20_	434.8	602.3 ± 6.4j	306 ± 16de	477 ± 26de	38.8 ± 0.5d	7421 ± 341de	2813 ± 62d	1725 ± 64de	0.383 ± 0.011cde	0.612 ± 0.008 fg
DI_30_N_30_	434.8	603.4 ± 5.9j	326 ± 12d	506 ± 24 cd	41.6 ± 0.6bc	7519 ± 336de	2870 ± 76d	1825 ± 60d	0.384 ± 0.011cde	0.636 ± 0.009d
DI_40_N_0_	382.7	568.6 ± 7.9m	171 ± 11k	223 ± 13k	30.9 ± 0.5k	4389 ± 356j	1426 ± 54h	742 ± 35j	0.333 ± 0.014j	0.520 ± 0.006m
DI_40_N_10_	382.7	572.8 ± 6.2m	235 ± 15hi	345 ± 20hi	35.7 ± 0.7gh	5412 ± 324i	1904 ± 53h	1089 ± 48i	0.357 ± 0.011hi	0.570 ± 0.009j
DI_40_N_20_	382.7	575.5 ± 5.9m	254 ± 10ghi	391 ± 14fgh	36.7 ± 0.5 fg	5895 ± 286gh	2099 ± 44f	1264 ± 34gh	0.358 ± 0.011hi	0.602 ± 0.007hi
DI_40_N_30_	382.7	576.3 ± 6.4l	276 ± 8efg	419 ± 15ef	38.5 ± 0.7e	5974 ± 299gh	2122 ± 54f	1289 ± 48g	0.359 ± 0.011hi	0.607 ± 0.007gh
DI_50_N_0_	330.6	515.6 ± 6.4o	134 ± 8l	155 ± 8l	29.8 ± 0.3k	3100 ± 312l	909 ± 50j	460 ± 35k	0.303 ± 0.014k	0.504 ± 0.008n
DI_50_N_10_	330.6	520.6 ± 7.6n	185 ± 12k	248 ± 15k	35.0 ± 0.5hi	3785 ± 312k	1226 ± 56i	646 ± 35j	0.332 ± 0.015j	0.527 ± 0.004m
DI_50_N_20_	330.6	523.4 ± 7.1n	196 ± 9jk	264 ± 13jk	35.4 ± 0.3gh	4041 ± 293jk	1327 ± 53hi	727 ± 33j	0.334 ± 0.013j	0.549 ± 0.008kl
DI_50_N_30_	330.6	522.7 ± 5.8n	197 ± 8jk	272 ± 13jk	36.6 ± 0.5 fg	4110 ± 273jk	1316 ± 48hi	728 ± 36j	0.325 ± 0.011j	0.552 ± 0.009kl

Values within a column for each variable followed by different letter/s are significantly different at *P* < 0.05 level. FI: full irrigation means irrigation equaled to 100% ETc; DI_10_:10% deficit irrigation means irrigation equaled to 90% ETc; DI_20_: 20% deficit irrigation means irrigation equaled to 80% ETc; DI_30_: 30% deficit irrigation means irrigation equaled to 70% ETc; DI_40_: 40% deficit irrigation means irrigation equaled to 60% ETc; DI_50_: 50% deficit irrigation means irrigation equaled to 50% ETc. N_0_ : 0 kg N ha^−1^; N_10_: 10 kg N ha^−1^; N_20_: 20 kg N ha^−1^; N_30_: 30 kg N ha^−1^.

**Figure 1:**
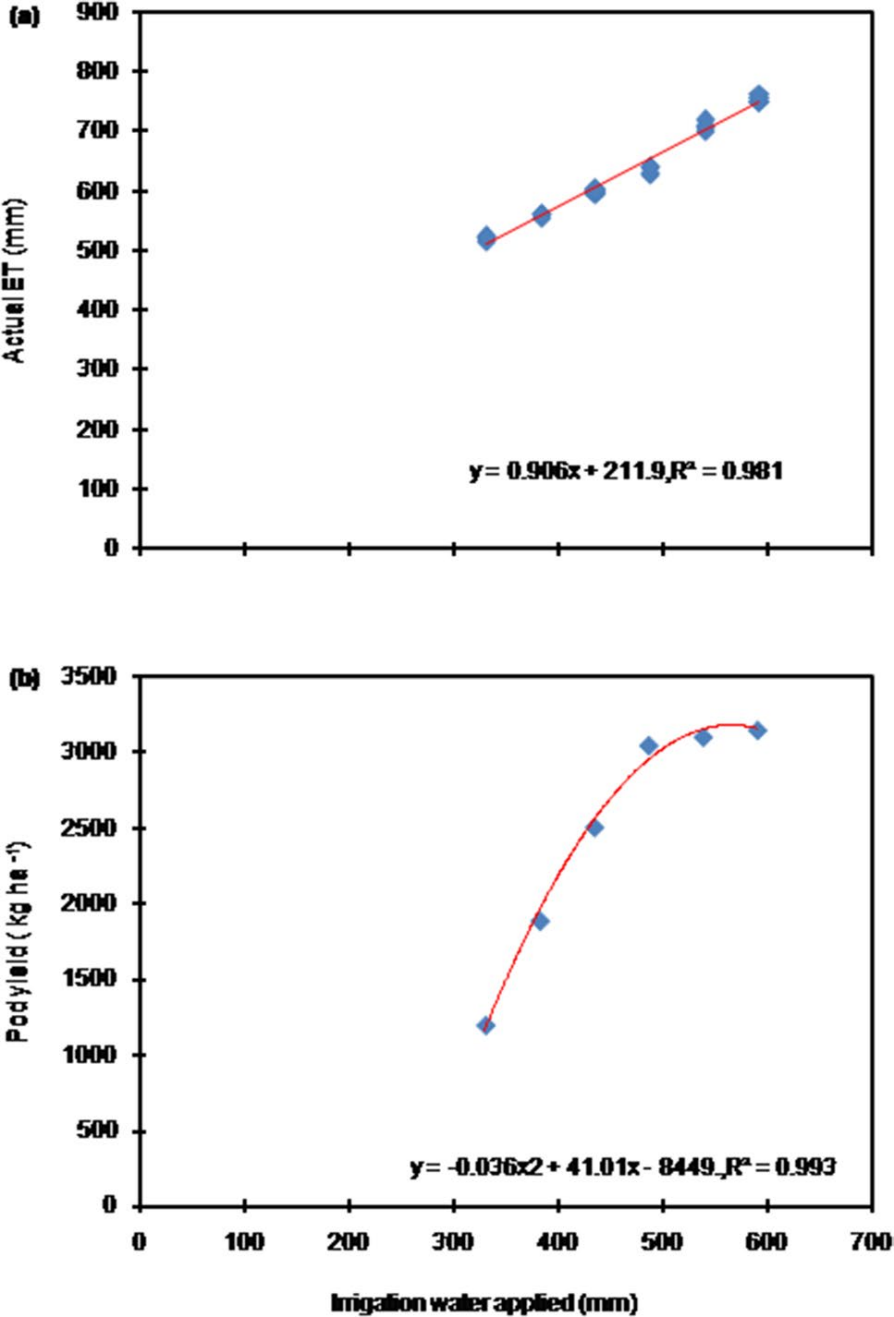
Relationship between irrigation water applied and AET (**a**) and AET and pod yield (**b**) of peanut during 2012–2014 at Bikaner, India. Each data point is average of 36 values. AET : actual crop evapotranspiration.

Relationship between AET and irrigation amount was linear and had a high (0.98) coefficient of determination (R^2^) (Fig. 1a). Slopes of the linear relationship of AET with irrigation water applied were found 0.90, suggesting that about 90% of applied water is converted to AET. Pod yield exhibited a quadratic relationship with amount of irrigation applied (Fig. 1b), implying that after a certain level of irrigation amount (~ 500 mm), a further increase in irrigation amount will not result into the same amount of increase in pod yield rather it will be less.

### Growth, yield and quality

**Figure 2:**
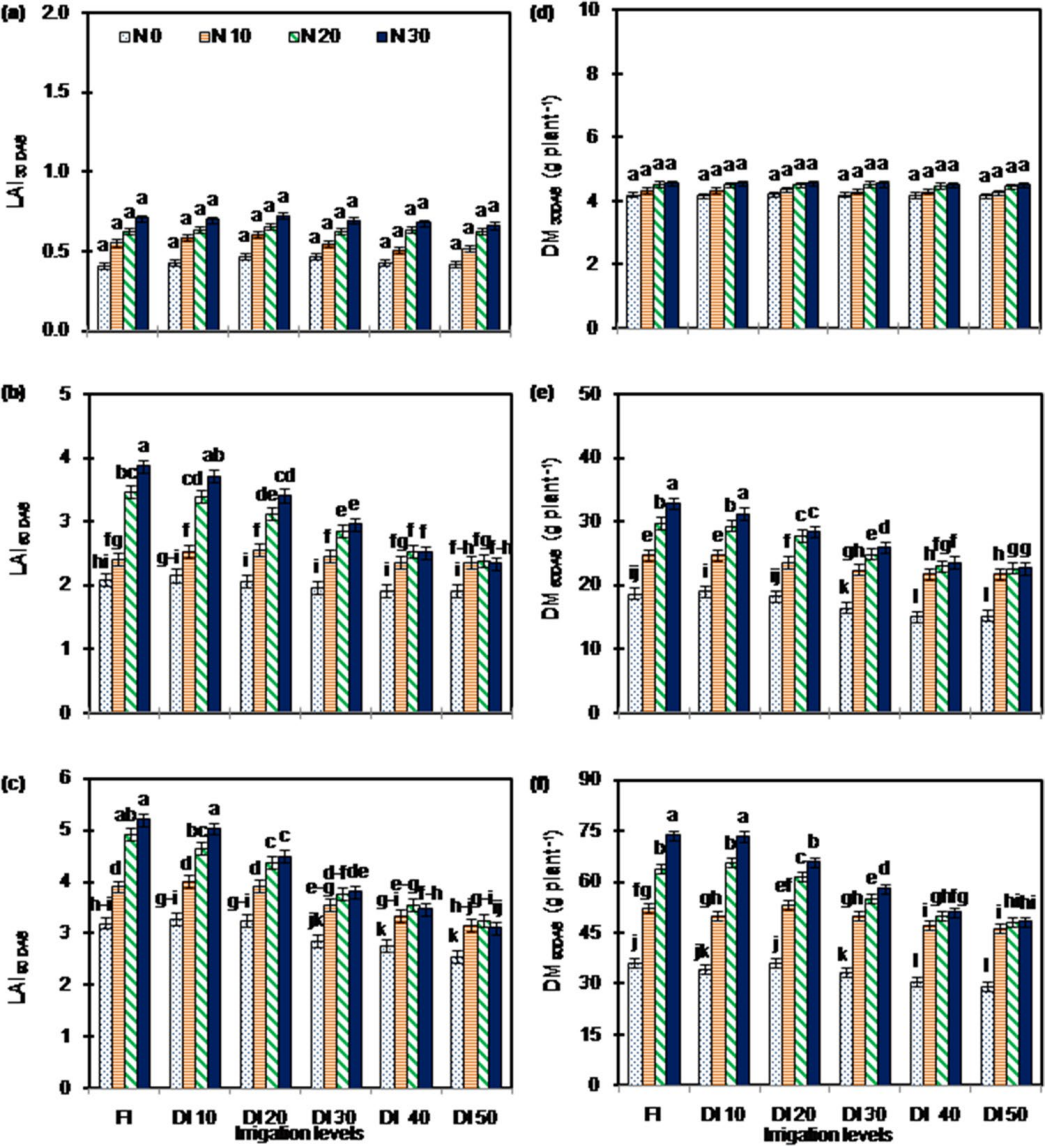
Leaf area index at (**a**) 30 DAS, (**b**) 60 DAS, (**c**) 90 DAS and dry matter production at (**d**) 30 DAS, (**e**) 60 DAS, (**f**) 90 DAS of peanut under different irrigation and N application rates. [Data points are mean ± standard error, n = 9 (3 × 3; 3 growing seasons and 3 replications per treatment)].Error bars are standard error. Bar followed by different letter/s are significantly different at *P* < 0.05 level according to LSD. LAI: leaf area index; DM: dry matter production; DAS : days after sowing. The details of treatments are given in Table 1.

The growth (leaf area index, dry matter production) (Fig. 2), yield components (pod number, kernel number, 100-kernel weight), biomass partitioning (pod harvest index, shelling fraction), yield (biomass yield, pod yield, kernel yield) (Table 1), and quality (Table 2) of peanut were significantly (*P* < 0.05) affected by irrigation levels, N application rates and their interaction.

**Table 2:** Content of oil and protein in kernel of peanut under different irrigation and nitrogen application rate treatments [Values are mean ± standard error, *n* = 9 (3 × 3; 3 growing seasons and 3 replications per treatment)].

Treatments	Oil content of kernel (g kg^−1^)	Protein content of kernel (g kg^−1^)
FIN_0_	4.66 ± 0.03ef	2.24 ± 0.01l
FIN_10_	4.92 ± 0.04c	2.37 ± 0.01j
FIN_20_	5.13 ± 0.06b	2.45 ± 0.02i
FIN_30_	5.19 ± 0.04ab	2.54 ± 0.02g
DI_10_N_0_	4.69 ± 0.04ef	2.25 ± 0.02l
DI_10_N_10_	4.97 ± 0.03c	2.37 ± 0.19j
DI_10_N_20_	5.13 ± 0.03b	2.47 ± 0.02i
DI_10_N_30_	5.21 ± 0.06ab	2.58 ± 0.02f
DI_20_N_0_	4.63 ± 0.04f	2.31 ± 0.02k
DI_20_N_10_	4.90 ± 0.05c	2.51 ± 0.01h
DI_20_N_20_	5.17 ± 0.04ab	2.61 ± 0.02e
DI_20_N_30_	5.26 ± 0.06a	2.74 ± 0.03b
DI_30_N_0_	4.43 ± 0.03g	2.24 ± 0.02l
DI_30_N_10_	4.75 ± 0.04de	2.54 ± 0.02g
DI_30_N_20_	4.91 ± 0.05c	2.70 ± 0.03c
DI_30_N_30_	4.87 ± 0.04 cd	2.79 ± 0.03a
DI_40_N_0_	4.48 ± 0.03g	2.25 ± 0.02l
DI_40_N_10_	4.62 ± 0.03f	2.39 ± 0.02j
DI_40_N_20_	4.67 ± 0.04ef	2.65 ± 0.02d
DI_40_N_30_	4.64 ± 0.04ef	2.75 ± 0.02b
DI_50_N_0_	4.29 ± 0.04h	2.23 ± 0.02l
DI_50_N_10_	4.40 ± 0.03gh	2.38 ± 0.02j
DI_50_N_20_	4.45 ± 0.03g	2.61 ± 0.02e
DI_50_N_30_	4.40 ± 0.02gh	2.62 ± 0.03e

Values within a column for each variable followed by different letter/s are significantly different at *P* < 0.05 level. The details of treatments are given in Table 1.

The growth, yield components and yield decreased with a reduction in irrigation and N application rates. Relative to FI, the DI_50_ had significant reduction in growth (24.2 to 30.1%) (Fig. 2), yield components (15.2 to 60.8%) and yield (55.9 to 67.6%) (Table 1). Averaged across years and irrigation levels, N_30_ enhanced yield (1.6 to 2.0-folds), yield components (1.3 to 2.0-folds), and growth (1.4 to 1.8-folds) compared to that of without N application (N_0_). The effects of N application rates on growth, yield components and yield were modified with irrigation levels. The N_30_ under FI and DI_10_ had greater (*P* < 0.05) growth and yield components than other treatments. Under DI_40_ and DI_50_, there was no significant (*P* > 0.05) difference in pod number and kernel number among N_30_, N_20_ and N_10_, but the values were greater (*P* < 0.05) than those of N_0_. The N_30_ under FI had greater (*P* < 0.05) biomass yield (11.0 to 237.9%) and pod yield (10.9 to 346.3%) than other treatments. The FIN_30_ and DI_10_N_30_ had greater (*P* < 0.05) kernel yield (7.4 to 481.1%) than other treatments. The N_30_ under FI, DI_10_ and DI_20_ had significantly greater pod and kernel yields than other N rates. The difference in yields among N_10_, N_20_ and N_30_ under DI_50_ were not significant, but yields were greater (*P* < 0.05) than those of N_0_.

The biomass partitioning measured in terms of pod harvest index and shelling fraction enhanced with a reduction in irrigation from FI to DI_20_; and further reduction in irrigation declined biomass partitioning (Table 1). The DI_20_ had greatest values for pod harvest index and shelling fraction. The N application enhanced both pod harvest index (4.4 to 9.5% greater than N_0_) and shelling fraction (10.2 to 18.1% greater than N_0_). The N_30_ under DI_20_ had significantly greater pod harvest index and shelling fraction than other treatments.

The quality attributes of kernel measured in terms of oil and protein contents significantly affected by irrigation levels, N application rates and their interactions (Table 2). Oil and protein content of kernels varied from 4.29 to 5.26 g kg^−1^ and 2.23 to 2.74 g kg^−1^, respectively. The oil contents in kernel declined with a reduction in irrigation and N application rates. The N_30_ under DI_20_ irrigation level had highest oil content followed by N_30_ under DI_10_ and FI irrigation levels. A significant increase in oil contents were recorded up to application of N_20_ under all irrigation levels. The protein content of kernel enhanced with a reduction in irrigation. Averaged across years and N rates, DI_30_ had highest protein contents in kernel followed by DI_20_, DI_40_, DI_50_, DI_10_, and FI. The DI_20_, DI_30_ and DI_40_ had greater (P < 0.05) protein content than those under other irrigation levels. The N application enhanced protein content in the kernel. The DI_30_N_30_ had greater (*P* < 0.05) protein content in kernel than other treatments.

### Water productivity and nutrient-use efficiency

**Figure 3:**
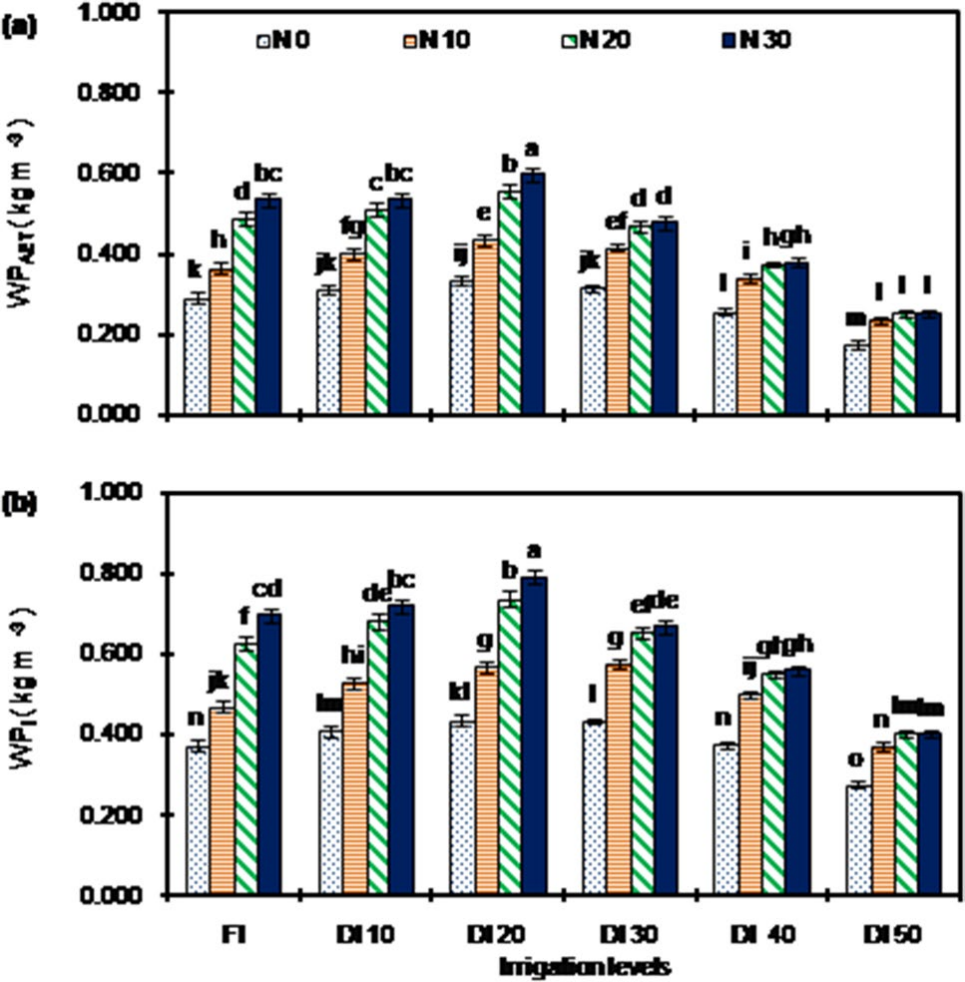
(**a**) WP_AET_ and (**b**) WP_I_ of peanut under different irrigation and nitrogen application rate treatments [Data points are mean ± standard error, n = 9 (3 × 3; 3 years, 3 replications)]. Bars are standard error. Bars having different letter/s are significantly different at *P* < 0.05 level according to LSD. AET : actual evapotranspiration; WP_AET_ : water productivity in terms of AET; WP_I_ : water productivity in terms of irrigation water applied. The details of treatments are given in Table 1.

**Figure 4:**
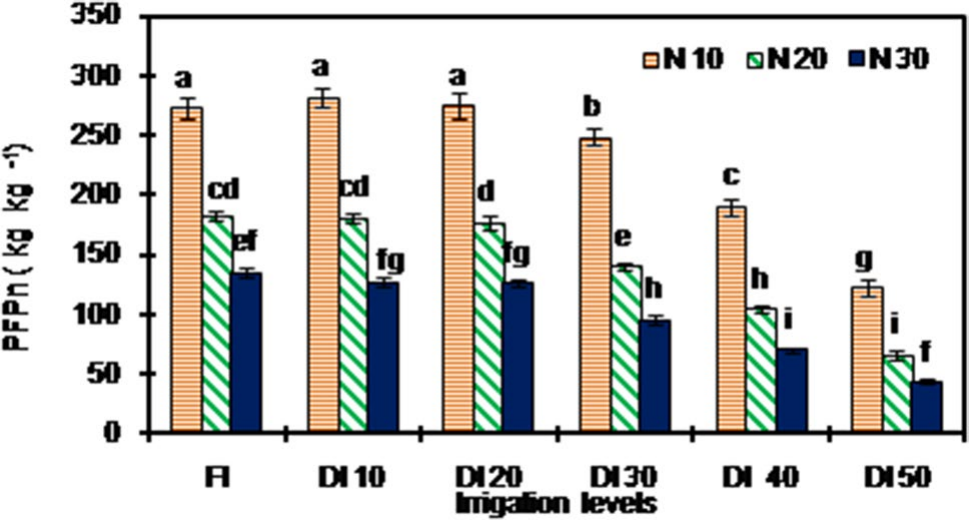
Nitrogen-use efficiency of peanut under different irrigation and nitrogen application rate treatments [Data points are mean ± standard error, n = 9 (3 × 3; 3 growing seasons and 3 replications per treatment)].Error bars are standard error. Bar followed by different letter/s are significantly different at P < 0.05 level according to LSD. PFP_n_ : partial factor productivity of N. The details of treatments are given in Table 1.

Irrigation levels, N rate and their interaction had significant (*P* < 0.05) effect on WP (WP_AET_: water productivity in terms of AET, and WP_I_: water productivity in terms of irrigation water applied) (Fig. 3), and NUE (Fig. 4). The WP_AET_ and WP_I_ varied from 0.176 to 0.597 kg m^−3^ and 0.275 to 0.791 kg m^−3^, respectively. The DI_20_N_30_ had highest WP which was significantly greater and had advantage to the tune up to 239.6% for WP_AET_ and up to 193.0% for WP_I_) over other treatments. Averaged across years and N rates, the WPs increased with a reduction in irrigation up to DI_20_. However, with further reduction in irrigation (DI_40_ and DI_50_) declined WPs. The DI_20_ had highest WP (WP_AET_: 0.481 kg m^−3^, WP_I_: 0.633 kg m^−3^) which was significantly greater (from 9.5 to 110.2% for WP_AET_ and from 8.3 to 74.8% for WP_I_) than other irrigation levels. The N application augmented WP, and N_30_ had 64.4 and 66.6% greater WP_AET_ and WP_I_, respectively than with N_0_.

The NUE measured in terms of partial factor productivity of N (PFP_n_) varied significantly among different treatments (Fig. 4). The PFPn declined with a reduction in irrigation and an increase in N application rates. The N_10_ under FI, DI_10_ and DI_20_ irrigation levels had similar PFPn, which was significantly greater than other treatments. Averaged across years and N application rates, the difference in PFPn among FI, DI_10_ and DI_20_ were not significant. The N_20_ and N_30_ treatments had 38.7 and 56.9% reduction in PFPn compared to N_10_.

### Economic benefits

The cost of cultivation varied considerably (from US $ 979.7 to US$ 1299.0) among different treatments (Table 3). The irrigation, N rates and their interaction significantly affected economic returns. The returns increased with an increase in irrigation and N application rates. The DI_40_ along with N_0_ and DI_50_ with all N rates had economic loss (negative net return).The FI, DI_10_ and DI_20_ had similar net return (from US $ 804 to 826 ha^−1^) which was significantly greater (from US $ 349.5 to 776.7 ha^−1^) than those in other irrigation levels. The N application enhanced returns. The FIN_30_ had greatest return, and the DI_50_N_0_ had lowest return.

**Table 3:** Cost and economic benefits of peanut under different irrigation and N application rates [Values are mean ± standard error, n = 9 (3 × 3; 3 growing seasons and 3 replications per treatment)].

Treatments	Cost of cultivation (US $ ha^−1^)	Gross return (US $ ha^−1^)	Net return (US $ ha^−1^)
FIN_0_	1299.0	1466.6 ± 48.3g	167.6 ± 36.2h
FIN_10_	1301.6	1836.7 ± 50.1e	535.2 ± 39.8f
FIN_20_	1303.0	2438.2 ± 43.6bc	1135.1 ± 32.7c
FIN_30_	1304.5	2683.6 ± 63.1a	1379.1 ± 55.2a
DI_10_N_0_	1234.5	1459.4 ± 37.9g	224.9 ± 28.4h
DI_10_N_10_	1237.0	1882.6 ± 41.2e	645.5 ± 37e
DI_10_N_20_	1238.5	2395.2 ± 42.6cd	1156.7 ± 31.9c
DI_10_N_30_	1239.9	2516.5 ± 64.6b	1276.7 ± 39.4b
DI_20_N_0_	1171.1	1398.1 ± 51.0g	227.1 ± 38.2h
DI_20_N_10_	1173.6	1819.4 ± 62.1e	645.8 ± 44.1e
DI_20_N_20_	1175.1	2318.9 ± 64.5d	1143.8 ± 46.2c
DI_20_N_30_	1176.5	2461.9 ± 65.2bc	1285.4 ± 52.3ab
DI_30_N_0_	1107.7	1267.6 ± 50.1h	159.9 ± 37.6h
DI_30_N_10_	1110.2	1658.7 ± 61.2f	548.5 ± 40.2ef
DI_30_N_20_	1111.7	1868.2 ± 54.8e	756.5 ± 41.1d
DI_30_N_30_	1113.1	1906.1 ± 70.9e	793.0 ± 43.2d
DI_40_N_0_	1043.1	985.0 ± 54.6i	-58.1 ± 20.9i
DI_40_N_10_	1045.7	1289.1 ± 55.4h	243.5 ± 41.5h
DI_40_N_20_	1047.1	1416.6 ± 48.3g	369.5 ± 36.2g
DI_40_N_30_	1048.6	1434.4 ± 56.9g	385.8 ± 37.5g
DI_50_N_0_	979.7	645.2 ± 48.6k	-334.6 ± 36.4j
DI_50_N_10_	982.3	847.5 ± 52.1j	-134.7 ± 39.0i
DI_50_N_20_	983.7	913.9 ± 50.0ij	-69.8 ± 27.5i
DI_50_N_30_	985.2	912.4 ± 46.4ij	-72.9 ± 24.8i

Values within a column for each variable followed by different letter/s are significantly different at *P* < 0.05 level. The details of treatments are given in Table 1.

### Comprehensive evaluation based on TOPSIS

The evaluation of peanut production under 27 treatments by using TOPSIS (Table 4) showed that the comprehensive benefit evaluation index (C_i_) varied from 0.164 to 0.880 with DI_20_N_30_ emerging as the best treatment followed by DI_10_N_30_ and DI_20_N_20_. The treatment with highest degree of deficit in irrigation and no nitrogen application (DI_50_N_0_) proved to be the poorest treatment. The DI_20_N_30_ had 4.2 to 73.8% greater C_i_ than in N_30_ with other irrigation levels. Compared to FI; DI_10_ and DI_20_ had 6.4 to 9.9% greater C_i_, but under DI_30_, DI_40_ and DI_50_ the C_i_ reduced by 10.4, 38.4 and 64.8%, respectively. The N_10_, N_20_ and N_30_ had 64.2, 124.4 and 135.5% greater C_i_ than that of with N_0_. The DI_40_, DI_50_ irrigation levels and N_0_ and N_10_ nitrogen application rates were not conducive for acquiring comprehensive benefits. The comprehensive benefit was positively related to pod yield, net return, oil content in kernel, and WP_I_; and negatively related to amount of irrigation water applied.

**Table 4:** TOPSIS analysis of irrigation water applied, yield, water productivity, oil content in kernel, and economic benefit for different irrigation and N application rate treatments for peanut.

Treatments	X_1_	X_2_	X_3_	X_4_	X_5_	D_*i*_^+^	D_*i*_^−^	C_*i*_	Rank
FIN_0_	0.034	0.010	0.040	0.028	0.051	0.084	0.035	0.296	19
FIN_10_	0.042	0.030	0.042	0.035	0.051	0.062	0.059	0.488	12
FIN_20_	0.056	0.065	0.044	0.047	0.051	0.030	0.098	0.766	6
FIN_30_	0.063	0.078	0.044	0.052	0.051	0.024	0.113	0.827	4
DI_10_N_0_	0.034	0.013	0.040	0.030	0.047	0.079	0.039	0.330	17
DI_10_N_10_	0.044	0.037	0.042	0.039	0.047	0.053	0.066	0.555	10
DI_10_N_20_	0.056	0.066	0.044	0.050	0.047	0.025	0.100	0.802	5
DI_10_N_30_	0.059	0.073	0.044	0.053	0.047	0.020	0.108	0.843	2
DI_20_N_0_	0.032	0.013	0.039	0.032	0.042	0.078	0.040	0.338	16
DI_20_N_10_	0.042	0.037	0.042	0.042	0.042	0.051	0.067	0.568	9
DI_20_N_20_	0.055	0.065	0.044	0.055	0.042	0.021	0.100	0.827	3
DI_20_N_30_	0.059	0.073	0.045	0.059	0.042	0.015	0.110	0.880	1
DI_30_N_0_	0.029	0.009	0.038	0.032	0.038	0.082	0.037	0.308	18
DI_30_N_10_	0.038	0.031	0.041	0.043	0.038	0.056	0.062	0.523	11
DI_30_N_20_	0.043	0.043	0.042	0.048	0.038	0.043	0.076	0.640	8
DI_30_N_30_	0.044	0.045	0.041	0.049	0.038	0.040	0.078	0.659	7
DI_40_N_0_	0.022	-0.003	0.038	0.028	0.033	0.097	0.026	0.214	22
DI_40_N_10_	0.029	0.014	0.039	0.037	0.033	0.076	0.044	0.366	15
DI_40_N_20_	0.032	0.021	0.040	0.041	0.033	0.068	0.052	0.435	14
DI_40_N_30_	0.033	0.022	0.040	0.041	0.033	0.066	0.053	0.443	13
DI_50_N_0_	0.014	-0.019	0.037	0.020	0.029	0.116	0.023	0.164	24
DI_50_N_10_	0.019	-0.008	0.037	0.028	0.029	0.102	0.027	0.209	23
DI_50_N_20_	0.020	-0.004	0.038	0.030	0.029	0.097	0.030	0.233	20
DI_50_N_30_	0.020	-0.004	0.038	0.030	0.029	0.097	0.029	0.231	21
Z^+^	0.063	0.078	0.045	0.059	0.029				
Z^−^	0.014	-0.019	0.037	0.020	0.051				
R	0.95*	0.98*	0.94*	0.94*	-0.72*				

X_1_, X_2_, X_3_, X_4_ and X_5_ represent normalized value of pod yield, economic benefit, kernel oil content, irrigation water productivity (WP_I_), and amount of irrigation water applied, respectively. Z^+^ and Z^−^ are the positive and negative ideal solutions, respectively. Di^+^ and Di^−^ are the distance between each alternative and positive and negative ideal solution, respectively. C_*i*_ is the comprehensive benefit evaluation index for different treatments. R is Spearman correlation coefficient between comprehensive benefit index rank and single attribute index rank and the asterisk (*) means that the R was significant at α = 0.05.

### Discussion

Shortage of water is the major constraints for limiting crop yield in arid and semi-arid areas^34,35^; and improving effective utilization of water is an urgent need for sustainable crop production in these areas^6,7,36^. Deficit irrigation (DI) has been emerging as an effective practice to improve WP, and saving of water^4–7^. Prior to this study, little information exist on productivity, resource use efficiency (WP and NUE), and quality for peanut under varying irrigation and N application rates. Our results showed that the studied parameters were determined by irrigation regimes and N rates and their interactions, and there were also significant trade-off between different parameters.

In the present study, the DI saved irrigation water (from 8.8 to 44.1%) and decreased crop water consumption (AET, from 6.1 to 31.0%) compared to FI (Table 1). This reduction in water saving and AET compromised yield; and magnitude of yield decline depended on reduction in irrigation. DI with > 20% reduction in irrigation (DI_30_, DI_40_ and DI_50_) caused significant reduction in yield compared to FI. An adequate production of dry matter and its translocation to sinks are major determinants of yield; and water-deficit strongly influences them^37–40^. The yield decline with a reduction in irrigation observed in this study could be attributed to decrease in dry matter production (Fig. 2d–f) and biomass partitioning (Table 1), which resulted in decrease in yield components (Table 1). Another reason for a reduction in yield with reduced irrigation application might be an adverse effect on pegging^39,40^. The pod yield and kernel yield exhibited less reduction than biomass yield under moderate DI (DI_20_) level, suggesting that moderate DI stimulated biomass partitioning (higher pod harvest index and shelling fraction) resulting in marginal reduction in pod and kernel yield under DI_20_ observed in the present study.

**Figure 5:**
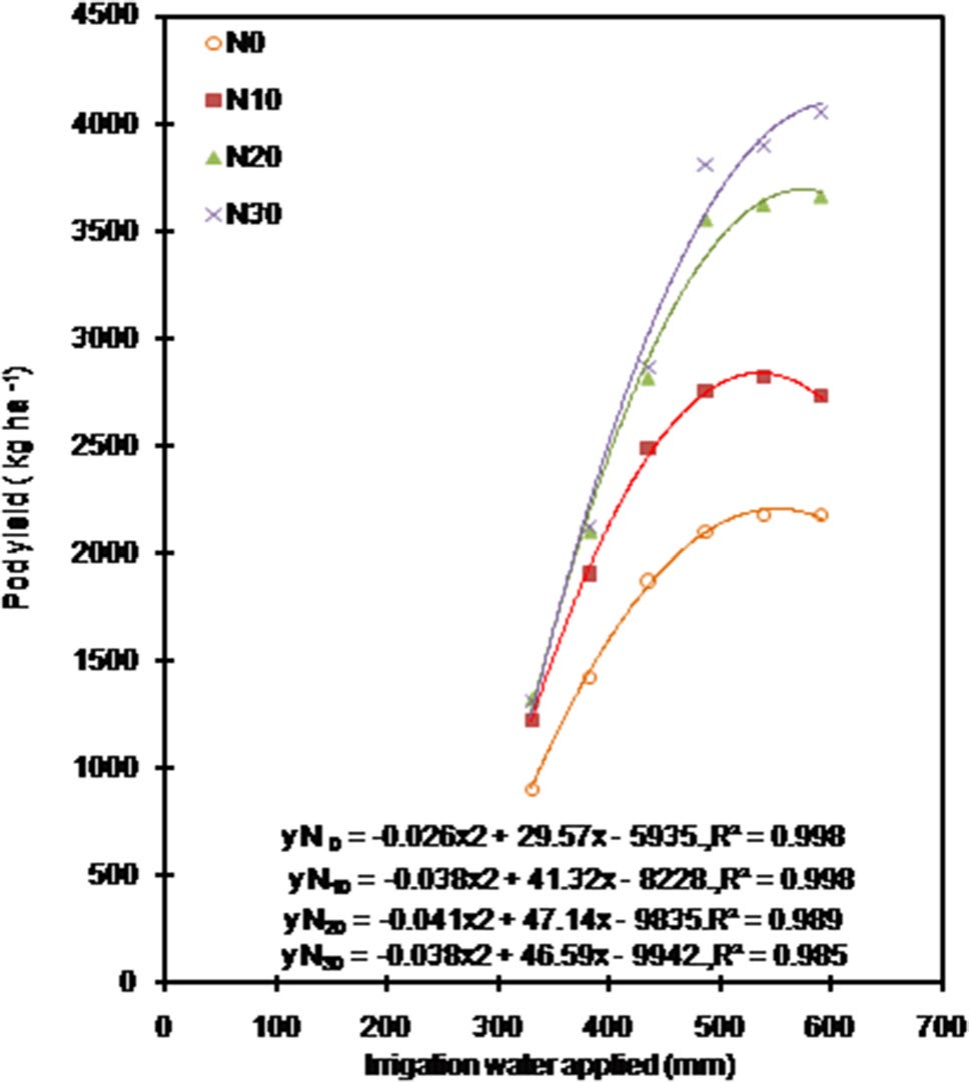
Relationship between irrigation water applied and pod yield of peanut under different N application rates during 2012–2014 at Bikaner, India. Each data point is average of 9 values. N_0_: 0 kg N ha^−1^; N_10_: 10 kg n ha^−1^; N_20_: 20 kg N ha^−1^; N_30_: 30 kg N ha^−1^.

In this study, N application increased yield (Table 1), and magnitude of which depended on irrigation levels (Fig. 5). Adequate N availability is essential for boosting peanut productivity^29,41^. The significantly higher yield with N-fertilization in our study was directly attributed to greater expression of yield contributing characters (Table 1) that were a result of the enhanced DM (Fig. 2d–f) and biomass partitioning (Table 1) due to N application. Our results showed that yield responses to N rates decreased with a reduction in irrigation. An increase in soil water availability positively affects mineralization and physical transport of N to roots, all of which increase plant-available N for uptake^42^. Limitation to mineralization and/or transport of N to roots surface in reduced water-supply conditions might be responsible for lack of yield response to higher N rates with a reduction in irrigation observed in the present study. The findings of our study provide direct evidence that application of N-fertilizer is effective to increase yield of peanut in the areas having soil with low content of N, and proper combination of irrigation and fertilizer can achieve the optimal coupling effect and optimize yield.

There are reported contrasting effects of DI for WP of peanut, ranging from an increase^43,44^ to a decrease^20,24,45^ of WPs. In the present study, WPs initially increased up to 20% reduction in irrigation (DI_20_); and further decreased with greater reduction in (DI_30_, DI_40_ and DI_50_) (Fig. 3). Relatively greater reduction in AET (15.9% reduction compared to FI) and amount of irrigation water application (17.6% reduction compared to FI) than reduction in pod yield (3.3% reduction compared to FI; Table 1) is responsible for higher WPs under moderate DI (DI_20_) as compared to FI observed in this study. Conversely, relatively greater reduction in yield (from 40.1 to 62.2% reduction in pod yield than FI) than reduction in AET (from 25 to 30% reduction than FI) and amount of irrigation water application might be responsible for lower WPs under severe DI (DI_40_ and DI_50_) compared to FI observed in this study. Besides, our results also demonstrated that N application enhanced WPs (Fig. 3), because of improved yield without much affecting crop water consumption (AET) (Table 1). Enhanced leaf area index (Fig. 2a–c) which lead to reduction in evaporation component of ET^13^ along with increase in yield due to enhanced dry matter production (Fig. 2d–f) and partitioning of dry matter to sink (evident by higher pod harvest index and shelling fraction) might be responsible for enhancement of WPs due to N application observed in this study. It has been reported that addition of N in N-deficient soil increased WPs, provided irrigation water is not limited^6,7,44^. Based on the above-mentioned benefits of both moderate DI and N application, the highest WPs were observed with DI_20_N_30_.

The NUE declined with a reduction in irrigation and an increase in N rates (Fig. 4).The N10 under FI, DI_10_ and DI_20_ had significantly greater NUE than other treatments. Adequate soil water positively affects availability (via mineralization and physical transport of ions in soil), uptake, and utilization of nutrients by plants and resulting in greater yield and NUE. Restricted soil water availability reduces the access of root to the mass flow of N leading to low uptake and utilization of N and a subsequent decrease in NUE^42,46^. The lower increase in yield than corresponding increase in N application rates is the major reason for declining in NUE with an increase in N rates observed in the present study. Our results showed that increasing N application rates, the WP increased (Fig. 3) and that of NUE declined (Fig. 4); suggesting the trade-off relationship between WP and NUE. But maintaining a higher WP with better yield at cost of NUE is more suitable for peanut production in water scarce areas like northwestern hot arid region of India.

Previous researchers reported contrary results regarding effect of water deficit on oil and protein content of kernel. There are reports that water-deficit had no effects^47^, and decline^27^ in oil content in kernel; no-consistent effect^47^, and increase^48^ in protein content in kernel of peanut. In this study, oil content of kernel declined, and protein content increased with a reduction in irrigation. Both oil and protein contents enhanced with an increase in N rates (Table 2). The enhanced oil and protein content of kernel due to N application is associated with the facts that adequate N supply is required for both protein and oil synthesis^28^. Inadequacy of photo-synthates (due to water and N-deficit induced reduction in leaf area and photosynthesis), and increased oxidation of fatty acids^49^ might be responsible for the reduction in oil content of kernel with a decrease in the irrigation and N application rates observed in the present study. Besides, water-deficit hinders the fat synthesis more than that of protein formation^50^ is responsible for greater reduction in oil content than that of protein content with a reduction in irrigation observed in this study.

Net return is an important criterion for evaluating irrigation and fertilizer management strategies for crop production. In this study, the net return increased with an increase in N rates under FI, DI_10_ and DI_20_ irrigation levels implying that increased cost with an increase in N rates in these irrigation levels balanced by value of improved yield due to the N application (Table 3). Cost incurred in all N rates under DI_50_ was not compensated by value of yield under these treatments and thus had economic loss.

Our results showed that WP, yield, quality, NUE and economic benefit were determined not only by application rates of irrigation and N, but also by their interaction. Besides, our results also demonstrated that there was trade-off relationship among these parameters; consequently it was difficult to obtain the maxima of WP, yield, quality, and saving of irrigation water simultaneously. In this study, maximum yield and net return were achieved when FI with N_30_ were applied, whilst WP and KOC were maximized with application of N_30_ under DI_20_ irrigation level. The TOPSIS was used to identify best alternative for optimizing WP, yield, quality, economic benefit, and saving of irrigation amount simultaneously. Since, the value of NUE is not possible to calculate in treatments involving N_0_ nitrogen application rate, the NUE was not considered for TOPSIS analysis in this study. Results of TOPSIS showed that moderate DI (DI_20_) in combination with N_30_ (DI_20_N_30_) could simultaneously achieve better yield, quality, return, irrigation water saving along with higher WP (Table 4).

## Conclusions

Three-year field experimentation on investigation of effects of irrigation and N regimes on WP, yield, quality, and economic return from peanut cultivation in northwestern arid India demonstrated that all the parameters were determined by both irrigation level and application rate of N, and there is an optimal level of N rate for each level of irrigation. Therefore, N application rate should be adjusted according to level of irrigation available. Results also clearly showed that it was not possible to maximize yield, quality, return and WP simultaneously. Rather, TOPSIS analysis showed that moderate DI (~ 20% deficit) along with application of N @30 kg ha^−1^ could balance among productivity, economic return, quality, and resource utilization for peanut cultivation in northwestern arid India.

## Materials and methods

### Study site and materials

Field experiments were conducted during rainy season (June–October) of 2012, 2013 and 2014 at the Regional Research Station of the ICAR- Central Arid Zone Research Institute, Bikaner, India (28º4′ N; 74º3′ E, 238.3 m above mean sea level). The experimental site is located in a hot arid climate and has mean annual maximum and minimum temperatures of 33.9ºC and 18.8ºC, respectively. The average annual rainfall is 263.5 mm, of which > 85% occurs during the southwest monsoon season (July to September). The mean weekly temperatures, rainfall and evaporation recorded during the experimentation periods at site are shown in supplementary Fig. S1.The soil of the site was loamy sand, Typic Torripssaments (USDA soil taxonomical classification), and contained 83.8% sand (0.02–0.2 mm), 4.8% silt (0.002–0.02 mm) and 11.4% clay (< 0.002 mm). The bulk density of soil was 1.57 g cm^−3^ and moisture content at field capacity was 0.157 m^3^ m^−3^. Top soil layer (0–20 cm) contains 1.3 g kg^−1^ organic carbon (Walkley–Black method), 44.1 mg kg^−1^available N (KMnO_4_oxidizable), 4.7 mg kg^−1^ available *P* (Olsen), 109.4 mg kg^−1^ available K (1 N Ammonium Acetate) with pH of 8.3 (1:2.5, soil : water).Peanut cultivar HNG-10 was used during experimentation.

### Experimental design

There were 24 treatments having six irrigation (I) levels and four nitrogen (N) fertilization rates. The experiment was conducted using a split-plot design with three replications for each treatment. Irrigation levels were laid out in main plots (9 m × 37 m) and N application rates were laid out in sub-plot (9 m × 7 m size)  (Supplementary Fig, SI 2). Irrigation amount equaled to 100% (representing no deficit and denoted by FI), 90% (denoted by DI_10_), 80% (denoted by DI_20_), 70% (denoted by DI_30_), 60% (denoted by DI_40_) and 50% (denoted by DI_50_) of crop evapotranspiration (ETc) were levels of irrigation. Levels of nitrogen (N) application rates were (i) zero N application (N_0_), (ii) 10 kg N ha^−1^ (N_10_), (iii) 20 kg N ha^−1^ (N_20_), and (iv) 30 kg N ha^−1^ (N_30_).The crop water demand (ETc) requirement was computed using the equation:

1 $${\text{ET}}_{{\text{c}}} = {\text{E}}_{{{\text{pan}}}} \times {\text{K}}_{{\text{p}}} \times {\text{K}}_{{\text{c}}}$$

where E_pan_ is pan evaporation (mm); K_p_ is pan coefficient (0.75); and K_c_ is crop coefficient, which varies for different growth stages of the crop^51^. Irrigation water was applied through sprinklers (double nozzle of 2.5 mm × 1.8 mm size with a discharge of 7.5 lpm at 2.5 kg cm^−2^ pressure, spaced at 10 m). A 70 mm pre-sowing irrigation was applied to each experimental plot. Subsequent irrigation water requirement (ET_c_) was computed as ET_c_ × irrigation efficiency (80%) as per irrigation levels. Irrigation was applied whenever cumulative ET_c_ reached approximately 45 mm. The evaporation was measured daily using USWB Class A pan evaporimeter located at 500 m away from the experimental site. Nitrogen was applied through urea fertilizer (46%N) as per N levels during land preparation prior to planting.

### Crop husbandry details

Soil was ploughed two times by disc harrow and then leveled. Phosphorus (P) was applied in the experimental plots during field preparation through one application of super phosphate (16% P_2_O_5_) @ 32 kg P_2_O_5_ ha^−1^. The crop was sown on 24^th^ June, 25^th^ June, and 27^th^ June during 2012, 2013, and 2014, respectively. Seeds were treated with Chlorpyriphos (O, O-Diethyl O-3, 5, 6-trichloropyridin-2-yl phosphorothioate) 20EC at the rate of 20 ml kg^−1^. Seeds were sown using a seed drill with a seed rate of 80 kg ha^−1^ and with a row spacing of 0.35 m. Weeds in the experimental plots were controlled manually.

### Measurements and analysis

Ten plants were randomly taken from each plot to measure the leaf area index (LAI) and dry matter (DM) at 30, 60 and 90 days after sowing (DAS). The leaf area was measured by a leaf area meter (Systronic India Ltd, Model 211), and LAI was calculated as the ratio of leaf area to harvested ground area^39^. The whole plants (leaf, stem, peg, and pod) were dried at 70 ºC to constant mass to determine DM. At maturity, total biomass yield and pod yield were recorded on central 4 m^2^ from each subplot after manual harvesting. The yield components, i.e., number of pod m^−2^, number of kernel m^−2^, and 100-kernel weight were recorded at maturity from each plot. Harvested material was sun dried for 7 days and threshed separately for each subplot to record pod yield. The pods were shelled manually to determine kernel yield. The pod harvest index was calculated by dividing pod yield with biomass yield. The shelling fraction was calculated as the ratio of kernel yield to pod yield.

For computing water consumed by the crop (AET), the soil profile water content was measured in 0–150 cm (with 20 cm increments for 0 to 100 cm, and 25 cm increments for 101 to 150 cm) soil by the thermo-gravimetric method. Volumetric soil water content was obtained by multiplying gravimetric water content by the corresponding bulk density and thickness of respective soil layers. The AET was calculated using the water balance method by monitoring the change in the soil water content prior to sowing and after harvest of crop:

2 $${\text{AET}} = {\text{P}}_{{\text{e}}} + {\text{I}} + {\text{UR}}{-}{\text{D}}_{{\text{w}}} {-}\Delta {\text{S}}$$

where AET is the actual crop evapotranspiration (mm), P_e_ is effective rainfall (mm), I is the irrigation (mm), U is upward capillary flow from ground water (mm), R is surface runoff (mm), D_w_ is drainage out of the root zone (mm), and S is the change in soil water storage in the 0–150 cm layer (mm), from planting to harvest of crops. As the groundwater level at experimental site is > 75 m deep, the U was ignored. The D_w_ was also considered zero as supported by the observation on negligible change in soil moisture content at soil depth > 120 cm. The R was also assumed negligible because the soil has good infiltration rate and each subplot was surrounded by 35 cm high bund. Therefore, the U, R and D_w_ were considered zero for calculating AET^7^.

The water productivity (WP) was calculated using the equations:

3 $${\text{WP}}_{{{\text{AET}}}} = {\text{PY/AET}}$$

4 $${\text{WP}}_{{\text{I}}} = {\text{PY/W}}_{{\text{I}}}$$

where WP_AET_ and WP_I_, is water productivity (kg m^−3^) in terms of AET and volume of irrigation water applied, respectively; PY is pod yield (kg ha^−1^), and AET is actual evapotranspiration (m^3^ ha^−1^) and W_I_ is volume of irrigation water applied (m^3^ ha^−1^).

The partial factor productivity of N (PFP_n_; kg kg^−1^) is an important measure for determining NUE. The PFP_n_ was calculated by using the following equation:

5 $${\text{PFP}}_{{\text{n}}} = {\text{PY/N}}_{{{\text{fi}}}}$$

where PY is pod yield (kg ha^−1^); N_fi_ is applied N as fertilizer in different treatments (kg ha^−1^).

The kernel oil content (KOC) was determined by the Soxhlet method^52^. Kernels were ground into a fine meal, and weighed quantity (2.5 g) of the meal was transferred to the thimble and extracted in 150 ml hexane in Soxhlet extraction assembly for 6 h. The solvent was then evaporated in an oven at 60ºC to a constant weight. The values of oil were calculated and expressed as g kg^−1^ (W W^−1^). To estimate kernel protein content, the N concentration was determined using the Kjeldahl method. A factor of 5.46 was used to convert N concentration into protein concentration^48^.

Economic benefit in terms of net return (NR) was calculated for each treatment. Components of cost of cultivation (CC) included investments of inputs (seed, fertilizer, pesticides), irrigation, labour and machinery cost for field operations (tillage, sowing, weeding, harvesting, threshing) and rental value of land. The cost of land was estimated using seasonal rental value of land prevailing in the region. Income from the sale of pod and straw yield was added to calculate gross return (GR). The income was calculated from current price prevailing in the study region. The NR was calculated using following equation:

6 $${\text{NR}} = {\text{GR}}{-}{\text{CC}}$$

where NR is net return ($ ha^−1^), GR is gross return ($ ha^−1^), and CC is cost of cultivation ($ ha^−1^).

### Multi-objective decision making and evaluation using TOPSIS

The technique for order preference by similarity to ideal solutions (TOPSIS) was used to identify best alternative for achieving better yield, quality, WP, economic benefit simultaneously for peanut following Deng et al.^32^. Calculation involves five sequential steps:

1. Constructing the original evaluation parameter matrix by using 3-year averaged values of parameters:
  7 $$X = \left( {x_{ij} } \right)_{n \times m} = \left[ {\begin{array}{*{20}c} {x_{11} } & {x_{12} } & {...} & {x_{1m} } \\ {x_{21} } & {x_{22} } & {...} & {x_{2m} } \\ {...} & {...} & {...} & {...} \\ {x_{n1} } & {x_{n2} } & {...} & {x_{nm} } \\ \end{array} } \right]$$
  where *x*_*ij*_ (*i* = 1,2,……..,*n* and *j* = 1,2,……,*m*) represents the *j*^th^ measurement (pod yield, kernel oil content, irrigation water productivity, economic benefit and irrigation amount used) from *i*^th^ treatment. In this study *n* = 24 and *m* = 5.
2. Construction of normalized decision matrix: For comparisons of attributes there is need of comparable scales which obtained by normalization. The vector normalization approach divides the rating of each attributes by its sum to calculate normalized value of *Z*_*ij*_ as defined in Eq. (8).
  8 $$Z_{ij} = W_{j} \frac{{x_{ij} }}{{\sqrt {\sum\limits_{i = 1}^{n} {x_{ij}^{2} } } }}$$
  The *Z*_*ij*_ is the normalized *x*_*ij*_ and *W*_j_ is the weight of the *j*th evaluated index. In this study, all evaluated parameter treated equally, therefore, *W*_*j*_ is 1.
  9 $$Z = \left[ {\begin{array}{*{20}c} {z_{11} } & {z_{12} } & {...} & {z_{1m} } \\ {z_{21} } & {z_{22} } & {...} & {z_{2m} } \\ {...} & {...} & {...} & {...} \\ {z_{n1} } & {z_{n2} } & {...} & {z_{nm} } \\ \end{array} } \right]$$
3. Determination of the positive (Z_max_, Z^+^) and negative (Z_min_, Z^−^) ideal solutions:
  10 $$Z_{j}^{ + } = \left\{ {\begin{array}{*{20}l} {\max \;z_{ij} ,{\text{Benefit}}\;{\text{type}}\;{\text{attribute}}} \hfill \\ {1 \le i \le n} \hfill \\ {\min \;z_{ij} ,{\text{Cost}}\;{\text{type}}\;{\text{attribute}}} \hfill \\ {1 \le i \le n} \hfill \\ \end{array} } \right.\quad i = 1,2, \ldots ,n;\quad j = 1,2, \ldots ,m$$
  11 $$Z_{j}^{ - } = \left\{ {\begin{array}{*{20}l} {\min \,z_{ij} ,{\text{Benefit}}\,{\text{type}}\;{\text{attribute}}} \hfill \\ {1 \le i \le n} \hfill \\ {\max \;z_{ij} ,{\text{Cost}}\,{\text{type}}\;{\text{attribute}}} \hfill \\ {1 \le i \le n} \hfill \\ \end{array} } \right.\quad i = 1,2, \ldots ,n;\quad j = 1,2, \ldots ,m$$
4. Calculation of Euclidean distance ($D_{i}^{ + }$ and $D_{i}^{ - }$) with Z^+^ and Z^−^:
  12 $$D_{i}^{ + } = \sqrt {\sum\limits_{j = 1}^{m} {\left( {z_{ij} - z_{j}^{ + } } \right)^{2} } }$$
  13 $$D_{i}^{ - } = \sqrt {\sum\limits_{j = 1}^{m} {\left( {z_{ij} - z_{j}^{ - } } \right)^{2} } }$$
5. Calculation of comprehensive benefit evaluation index (${C}_{i}$) for all treatments:
  14 $$C_{i} = \frac{{D_{i}^{ - } }}{{\left( {D_{i}^{ + } + D_{i}^{ - } } \right)}}$$
  where 0 ≤ *C*_*i*_ ≤ 1 When *C*_*i*_ is closer to 1, the peanut had better comprehensive benefit in terms of yield, quality, net return and water productivity.

### Statistical analysis

Data were statistically analyzed by ANOVA according to a split-plot design using Statistical Analysis System software. Data were subjected to Bartlett test for testing homogeneity of variance. The uniformity in error variance was not significant. The year × irrigation levels or year × N rate treatment effects on most of the parameters were not significant. Hence, the data were pooled and presented across the years. The means were compared using the least significant difference (LSD) test at 0.05 probability level.

## Supplementary Information

Supplementary Information 1.

## Data Availability

Data generated or analyzed during this study are included in this article (both as main file and supplementary information) and are available from corresponding author on request.
