# Supplementary material for: Optimization of deficit irrigation and nitrogen fertilizer management for peanut production in an arid region
Source: Sci Rep. 2021 Mar 9;11:5456. doi: 10.1038/s41598-021-82968-w (PMC7943784; doi:10.1038/s41598-021-82968-w)
Supplement: Supplementary file 1 — Supplementary Information 1. [file 41598_2021_82968_MOESM1_ESM.pdf]

Optimization of deficit irrigation and nitrogen fertilizer management for peanut production in an arid region

Vijay Singh Rathore[1*], Narayan Singh Nathawat[1], Seema Bhardwaj[1], Bhagirath Mal Yadav[1], Mahesh Kumar[2],Priyabrata Santra[2], Praveen Kumar[2], Madan Lal Reager[3], Narendra Dev Yadava[1] & Om Prakash Yadav[2]

[1]ICAR- Central Arid Zone Research Institute, Regional Research Station, Bikaner – 334004, India.

[2]ICAR- Central Arid Zone Research Institute, Jodhpur – 342003, India.

[3]KVK, Swami Keshwanand Rajasthan Agricultural University, Bikaner – 334006, India.

*Correspondence and request for materials should be addressed to V.S.R. (email: rathoreiari@yahoo.co.in)

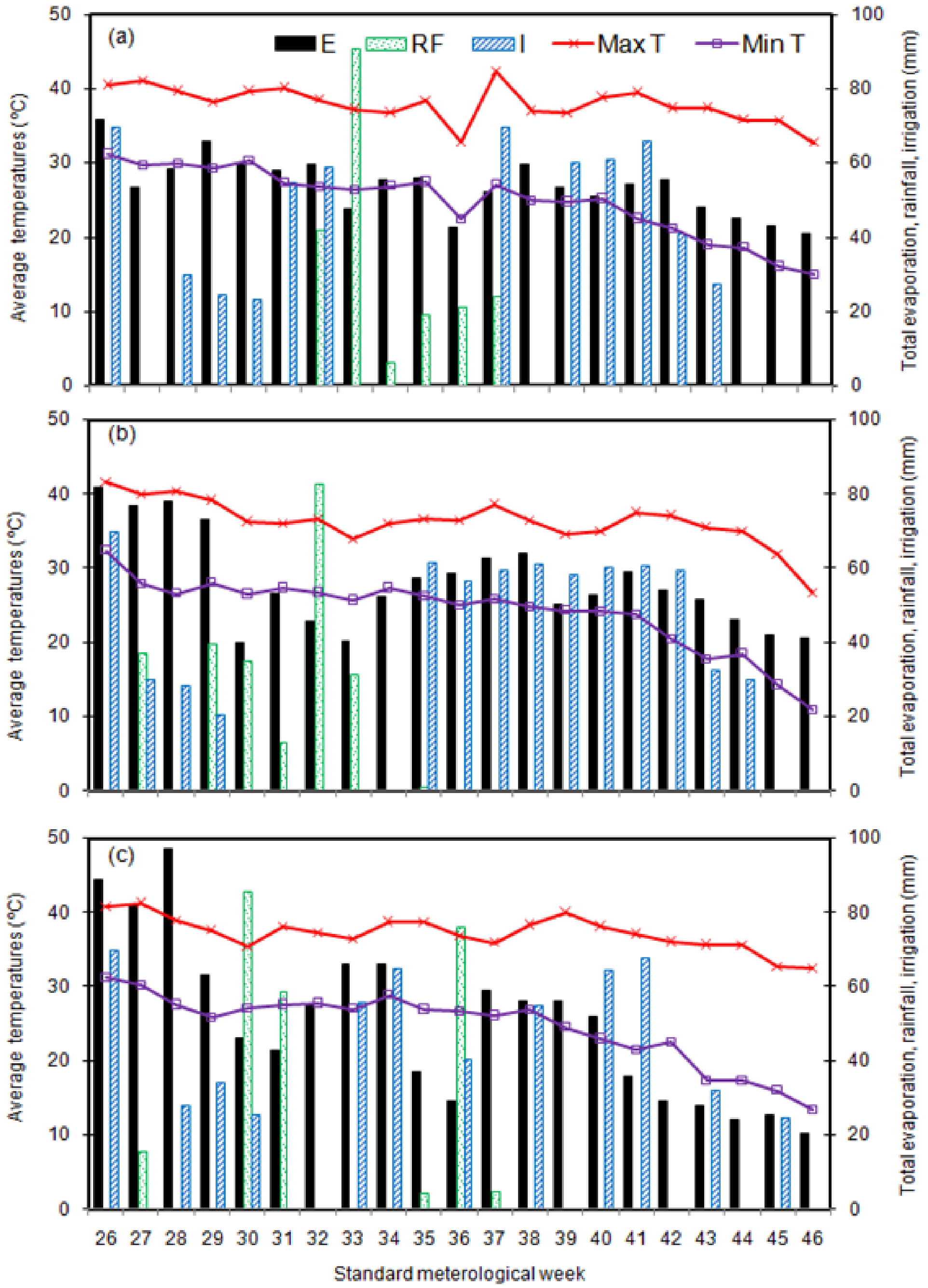

Fig. S1. Temperature, rainfall, evaporation and irrigation amount applied to FI treatment during peanut growing season in 2012 (a), 2013 (b) and 2014 (c) at Bikaner, India. E: Evaporation; RF: rainfall; I: amount of irrigation applied; Max T: average maximum temperature; Min T: average minimum temperature.

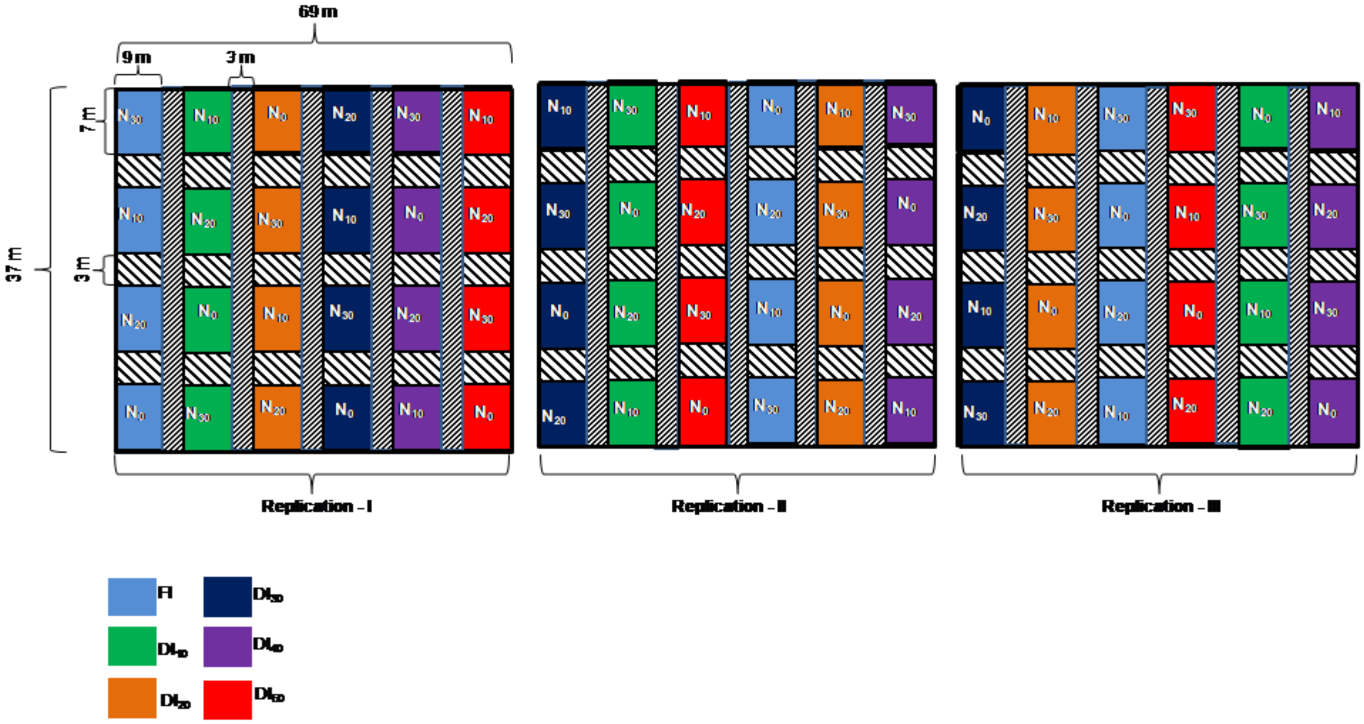

Fig. SI 2. Layout of experiment. FI: full irrigation means irrigation equaled to 100% ETc; DI10:10% deficit irrigation means irrigation equaled to 90% ETc; DI20: 20% deficit irrigation means irrigation equaled to 80% ETc; DI30: 30% deficit irrigation means irrigation equaled to 70% ETc; DI40: 40% deficit irrigation means irrigation equaled to 60% ETc; DI50: 50% deficit irrigation means irrigation equaled to 50% ETc. N0 : 0 kg N ha^{-1}; N10: 10 kg N ha^{-1}; N20: 20 kg N ha^{-1}; N30: 30 kg N ha^{-1}.
